# Proliferation of enterotoxigenic *Escherichia coli* strain TW11681 in stools of experimentally infected human volunteers

**DOI:** 10.1186/s13099-018-0273-6

**Published:** 2018-10-16

**Authors:** Oda Barth Vedøy, Kurt Hanevik, Sunniva Todnem Sakkestad, Halvor Sommerfelt, Hans Steinsland

**Affiliations:** 1 Department of Clinical Science, Faculty of Medicine, University of Bergen, Bergen, Norway; 2 Norwegian National Advisory Unit on Tropical Infectious Diseases, Department of Medicine, Haukeland University Hospital, Bergen, Norway; 3 Centre for Intervention Science in Maternal and Child Health (CISMAC), Department of Global Public Health and Primary Care, University of Bergen, Bergen, Norway; 4 Norwegian Institute of Public Health, Oslo, Norway; 5 Centre for International Health, Department of Global Public Health and Primary Care, University of Bergen, Bergen, Norway; 6 Department of Biomedicine, University of Bergen, Bergen, Norway

**Keywords:** Human volunteers, Experimental infection, Diarrhea, Enterotoxigenic *Escherichia coli*, Heat-stable enterotoxin, Controlled human infection model, Feces, Flow cytometry, Real-time polymerase chain reaction, Genomic DNA purification

## Abstract

**Background:**

As part of the effort to develop an enterotoxigenic *Escherichia coli* (ETEC) human challenge model for testing new heat-stable toxin (ST)-based vaccine candidates, a controlled human infection model study based on the ST-producing ETEC strain TW11681 was undertaken. Here, we estimate stool TW11681 DNA concentration and evaluate its association with dose, clinical symptoms, and with levels of antibodies targeting the CfaB subunit of the ETEC Colonization Factor Antigen I and the *E. coli* mucinase YghJ. Nine volunteers ingested different doses of the strain and were subsequently followed for 9 days with daily stool specimen collection and clinical examination. Stool DNA was purified by using a newly developed microplate-based method, and DNA originating from TW11681 was quantified by using a probe-based quantitative PCR assay. Antibody levels against CfaB and YghJ were measured in serum collected before and 10 and 28 days after TW11681 was ingested by using a bead-based flow cytometry immunoassay.

**Results:**

For 6 of the 9 volunteers, the stool TW11681 DNA concentration increased sharply a median 3.5 (range 2–5) days after dose ingestion, peaking at a median of 5.4% (range 3.3–8.2%) of the total DNA in the specimen. The concentration then fell sharply during the subsequent days, sometimes even before the onset of antibiotic treatment. The size or timing of these proliferation peaks did not seem to be associated with the number of TW11681 bacteria ingested, but the 2 volunteers who developed diarrhea and all five who experienced abdominal pains or cramps had these peaks. The 3 volunteers who did not have the proliferation peaks experienced fewer symptoms and they generally had relatively low CfaB- and YghJ-specific antibody levels before ingesting the strain and subsequently weaker responses than the other volunteers afterwards.

**Conclusions:**

Since the lack of proliferation peaks appears to be associated with fewer clinical symptoms and lower serum antibody responses to virulence factors of the infecting strain, it may be important to account for proliferation peaks when explaining results from controlled human infection model studies and for improving the accuracy of protective efficacy estimates when testing new ETEC diarrhea vaccine candidates.

## Background

Infections with enterotoxigenic *Escherichia coli* (ETEC) are a common cause of childhood diarrhea in low- and middle-income countries (LMICs) [1], as well as of diarrhea among travelers to these countries [2]. ETEC has emerged multiple times from the *E. coli* population through acquisition of plasmid genes encoding the large and highly immunogenic heat-labile toxin (LT) and/or the small and non-immunogenic heat-stable toxin (ST) [3, 4]. ETEC colonizes parts of the small intestine where it attaches itself to the intestinal wall cell surface, and most ETEC also produce colonization factors, which are surface appendages that help to stabilize the attachment [5]. Secreted ST binds to the guanylate cyclase-C receptor on the epithelial cell surface, which leads to increased secretion of salts into the intestinal lumen, which again drives diarrheal symptoms [6].

ETEC that produce ST, either alone or in combination with LT, are among the most important contributors to moderate and severe diarrhea among LMIC children, in addition to rotavirus, *Cryptosporidium* and *Shigella* [1]. Symptomatic infections with ST-producing ETEC also appear to be associated with an increased case fatality risk [1]. Efforts to develop effective ETEC vaccines are mainly focused on inducing anti-colonizing immunity through immunization with the main ETEC colonization factor antigens [7]. So far no effective vaccines have been produced, but several vaccine candidates are under development [8]. More recently, efforts have also been made to develop vaccines based on ST itself [8, 9].

Human ETEC may produce one of two close to identical variants of ST called porcine ST (STp) and human ST (STh). The ST-based vaccine development effort is primarily focused on STh since STh-producing ETEC are arguably a substantially more important cause of diarrhea among young LMIC children [10, 11].

To test the efficacy of ST-based vaccines, there is a need to develop a vaccine challenge model that allows for measuring the vaccines’ protection against diarrhea when volunteers are experimentally infected with strains that only produce ST. While controlled human infection models based on strains producing both ST and LT are available [12, 13], these strains may not be useful in vaccine challenge models for testing ST-based vaccines since the activity of LT could mask the effects of otherwise protective anti-ST immune responses. As part of the process to evaluate whether ETEC strain TW11681 could be suitable for use in a vaccine challenge model, we monitored the changes in TW11681 concentration in the stools of 9 volunteers experimentally infected with this strain and investigated whether changes in concentration were associated with differences in clinical symptoms and immune responses.

## Results

**Fig. 1:**
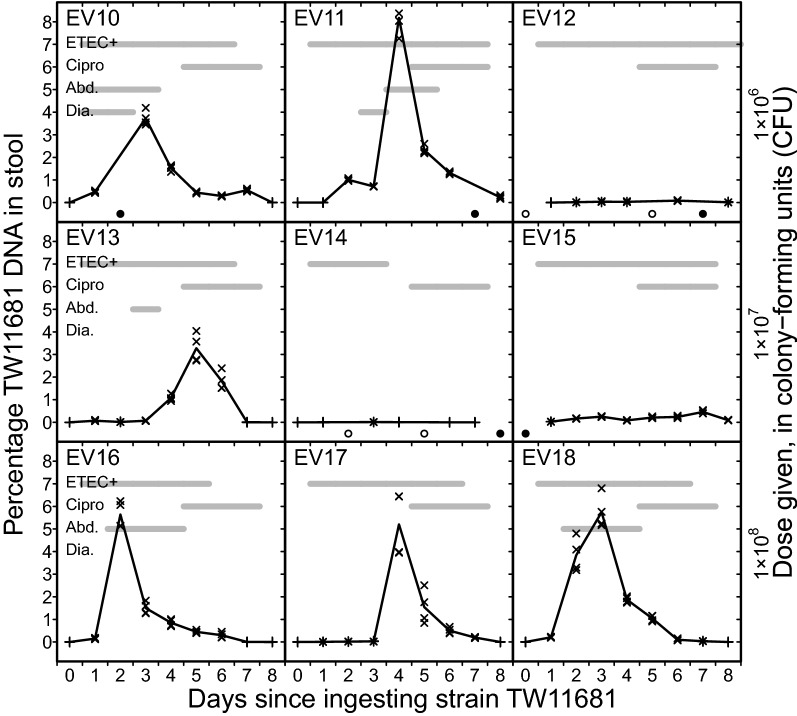
Percent DNA from TW11681 in stools from experimentally infected human volunteers. Four replicates, two of which were based on tenfold diluted template DNA, were performed on DNA isolated from one stool specimen from each day from each volunteer. Results are presented as the weight percentage of the DNA isolated from the specimen that originated from TW11681. Open (○) or closed (●) circles at the bottom of each graph indicate that no stools were passed, or that passed stools were not analyzed, respectively, on the given day. Crosses (×) or plusses (+) show the estimated TW11681 DNA percentages for individual qPCR runs where the results fell inside or outside the linear dynamic range of the assay, respectively. Results from all replicates are plotted, but some individual points may not be discernible due to overlap. The line-graphs represent the average percentage of all four replicates. The gray horizontal bars indicate which days the volunteers had ETEC microbiologically detected in their stool samples (“ETEC+”), were receiving ciprofloxacin treatment (“Cipro”), had abdominal pains or cramps (“Abd.”), or had diarrhea (“Dia.”)

Nine volunteers were followed for 9 days starting from the time of ingesting 1 × 10^6^, 1 × 10^7^ or 1 × 10^8^ colony-forming units (CFUs) of TW11681, and stool samples were successfully collected from 72 of these 81 volunteer-days (Fig. 1). Stool samples were not obtained on 4 volunteer-days (2 volunteers) because no stools were passed, while we failed to collect samples from passed stools on 5 volunteer-days (5 volunteers). Two of the 9 volunteers developed diarrhea, both of which were mild episodes, while five experienced mild or moderate abdominal pain or cramps (Fig. 1). Other observed signs and symptoms were few and mild. All volunteers started ciprofloxacin treatment 5 days after ingesting the dose.

Each volunteer cumulatively passed a median 1421 g (Interquartile range 1117 g, 1812 g; Range 780 g, 2114 g) of stools during the 9 day period, and all passed stools were grade 1 and 2 (formed), except for the stools passed during the two diarrheal episodes, which were grade 3 (viscous opaque liquid or semiliquid). ETEC was microbiologically detected in daily passed stool specimens for up to a median of 6 (range: 3–8) days after ingesting the dose (Fig. 1). The microbiological analyses yielded ETEC-negative results on 16 of the 81 volunteer-days, and stools from 13 of these days were found positive for TW11681 DNA in at least one qPCR replicate, albeit usually at a low percentage; the median of the mean of the TW11681-positive reactions was 0.006% (range: 0.001%–0.550%). Since the microbiological analyses detect mainly viable ETEC, some of the TW11681 DNA detected by qPCR may have originated from non-viable cells.

### qPCR assay validation

In the stool specimens collected on the day of infection, the O19 polymerase gene could either not be detected in the qPCR assay or was present in small amounts. This suggests that the volunteers were not extensively colonized with other O19 *E. coli* strains prior to ingesting the dose, and that most of the qPCR signals we observe can therefore be attributed to TW11681. The standard curve based on the positive control dilution series had acceptable curve fitting parameters (97% PCR efficiency and a mean squared error of 0.01), the assay has a linear dynamic range for detecting between 13 and 132,000 TW11681 genomes, and none of the no-template controls showed signs of amplification.

We found no indication that any of the qPCR results were affected by PCR inhibitors in the template DNA. Of the sample reactions that were based on the 0.1 ng/µl template DNA, 84, representing 43 different samples, gave results that fell within the dynamic range of the assay. The median within-sample quantitation cycle (Cq) difference between reactions based on 0.1 ng/µl and 1.0 ng/µl template DNA for these 43 samples was 3.10, the differences looked normally distributed (Shapiro–Wilk Normality Test *p* value: 0.22; skewness coefficient − 0.51), and there were no clear outliers. If the ETEC quantitation estimates presented here were substantially biased by PCR inhibitors from the template DNA preparations, the differences in Cq estimates between the two template concentrations would probably be substantially more skewed towards no Cq difference.

### TW11681 proliferation and association to clinical symptoms

For each stool specimen, we isolated DNA and quantified the number of TW11681 genomes by qPCR. The estimated TW11681 concentration in a stool specimen is represented by percent estimated weight of TW11681 genome DNA relative to the weight of all DNA from the specimen. The main weight of other DNA in the stool specimen probably originates from the remaining gut microbiota and from epithelial cells that has been exfoliated from the large intestine [14]. Assuming the production of these other cell populations remains relatively stable during the follow-up period, the changes in TW11681 DNA concentration should reflect actual changes in stool TW11681 proliferation during the infection.

As seen in Fig. 1, there were two main patterns for changes in the stool TW11681 DNA concentration. Six volunteers (EV10, EV11, EV13, EV16, EV17, and EV18) had a rapid and substantial increase and subsequent decline in TW11681 DNA concentration, with mean maximum concentration at 5.3% (range 3.3–8.2%) a median 3.5 (range 2–5) days after dose ingestion. The remaining 3 volunteers (EV12, EV14, and EV15) had low TW11681 DNA concentrations (< 0.5%) throughout the follow up period. The size of the TW11681 proliferation peaks did not seem to increase with inoculum size. Both volunteers who developed diarrhea and all five who experienced abdominal pain or cramps had these peaks, but the peaks did not necessarily coincide with the onset of symptoms. For example, EV11’s diarrheal episode and EV13’s episode with abdominal pain or cramps was over even before the TW11681 DNA concentration peaked. Notably, for EV10, EV16, and EV18, the rapid decline in the TW11681 DNA concentration started before onset of the antibiotic treatment.

### Immune responses

**Fig. 2:**
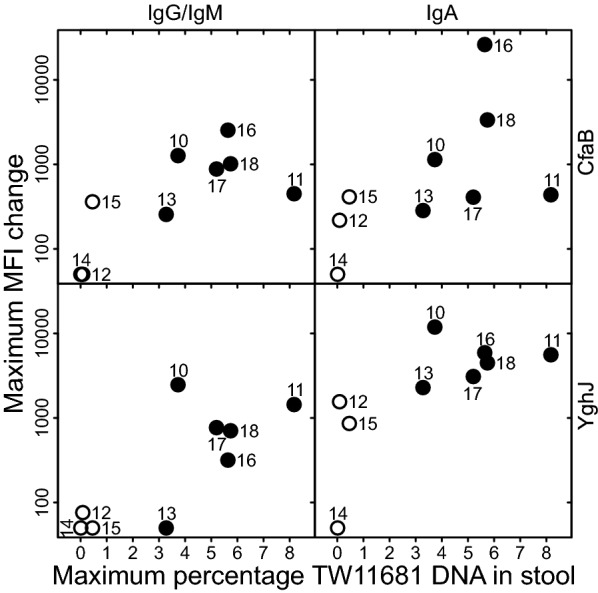
Maximum changes in CfaB- and YghJ-specific serum IgA and IgG/IgM responses. The numbers represent each volunteer’s study ID number (e.g. “10” = “EV10”). Open circles (○) represent the three volunteers who did not have TW11681 proliferation peaks in their stools, while closed circles (●) represent the six who did. The Y-axis values are plotted logarithmically and represent the maximum values of the median fluorescence intensity (MFI) of assay results based on serum collected 10 and 28 days after infection, minus the MFI of assay results based on the serum collected before the volunteer had ingested the dose. Prior to this calculation, MFI of assays results based on the negative controls were subtracted from all values

All volunteers except EV14 seemed to develop some serum antibody responses to the CfaB subunit of the ETEC Colonization Factor Antigen I (CFA/I) and the *E. coli* mucinase YghJ (Fig. 2). The three volunteers who did not have the TW11681 proliferation peaks (EV12, EV14, and EV15) had low levels of pre-existing CfaB- and YghJ-specific serum antibodies, similar to most of the other volunteers, but tended to have lower increases in antibody levels to these proteins than the other volunteers. Of the 6 volunteers who had TW11681 proliferation peaks, EV13 had the smallest peak as well as the lowest CfaB- and YghJ-specific serum antibody level increases (Fig. 2).

## Discussion

We found that the volunteers who were experimentally infected with ETEC strain TW11681 either had or had not a sharp and substantial increase in stool TW11681 excretion during follow-up, and that the substantially increased excretion sometimes dropped even before onset of the antibiotic treatment. The lack of these proliferation peaks tended to correlate with fewer clinical signs and symptoms, as well as lower increases in TW11681 virulence factor-specific serum antibody levels. These findings are relevant for the efforts to understand the dynamics of human ETEC infections, as well as for interpreting results from controlled human infection model studies and vaccine challenge trials.

Proliferation peaks similar to those observed in the current study has previously been observed by Pop et al. [15]. They performed 16S rRNA gene sequencing on DNA from daily collected stools specimens from 12 volunteers experimentally infected with ETEC strain H10407 and estimated the proportion of all bacteria in the stool specimen that was *E. coli*. In that study, all 7 volunteers who developed diarrhea had *E. coli* proliferation peaks, and all seven peaks were larger than those seen in the present study, with *E. coli* representing up to 78% of the bacterial population. In contrast to the present study where the proliferation peaks were found in both volunteers with and without diarrhea, *E. coli* proliferation peaks were not seen among the 5 volunteers who did not develop diarrhea.

The large difference in proliferation peak sizes between these two studies could, of course, be a result of differences in methods to estimate the strain concentrations, but it could also be attributed to whether or not the specimens were collected during diarrheal episodes and the severity of these episodes. In the present study, experimental infection with TW11681 only resulted in two mild diarrheal episodes during which the stool consistency only briefly reached grade 3 (viscous opaque liquid or semiliquid). Infections with H10407 tend to give longer and more severe episodes of grade 4 and 5 (watery) stools [12], and we expect the larger proliferation peaks they observed are mainly a result of the diarrhea purging the colon of other bacteria, leaving the analyzed specimens comprised mainly of H10407 cells that have been secreted from the small intestine. This explanation is supported by results from a study by Lindsay et al. where qPCR quantitation was used to investigate the correlation between stool ETEC quantities and having diarrhea. They found a higher concentration of ETEC in diarrheal than in non-diarrheal specimens that had been collected from the same volunteer on the same day [16].

Including qPCR results from watery diarrhea specimens in studies like these will probably complicate direct comparison of results, but the variation in peak size may turn out to be biologically less important than the actual presence of these peaks. In the current study, for example, peak presence, but not peak size, tended to be correlated with more symptoms and higher levels of serum antibody responses to the TW11681 virulence factors. The observation that symptoms tended to precede the stool proliferation peaks could indicate that the increased production of TW11681 that probably occurs in the small intestine is responsible for the symptoms, but that it takes time for the cells to pass through the colon.

Since a lack of a proliferation peak appears to be correlated with fewer symptoms, it may be advisable to perform strain proliferation monitoring of the volunteers when testing the efficacy of new vaccines against ETEC diarrhea. If a vaccinated volunteer does not develop a proliferation peak after being infected with the challenge strain, any lack of diarrhea may be a consequence of suboptimal colonization rather than be attributable to protection by the vaccine candidate.

Finally, we developed a new microplate-based DNA purification method for this project in order to obtain consistently clean DNA from a large number of stool specimens without having to work with individual tubes. Although both manual and automated microplate-based DNA purification methods and kits already exist, we find that these tend to be less effective at isolating DNA from Gram-positive than from Gram-negative bacteria in stools and that the purity of the DNA often varies. For the presented method, DNA is released from cells in the stools through treatment with heat, soaps, enzymes, and mechanical shearing, and subsequently purified by first salting-out proteins and soaps, precipitating with salts and alcohol, and crowding onto magnetic beads before a final rinse. We believe the combination of the three purification steps ensures that the purified stool DNA will be consistently sufficiently clean for use in PCR assays.

## Conclusions

We found that human volunteers experimentally infected with ETEC strain TW11681 either had a substantial and often self-limiting increase or no clear increase in stool TW11681 excretion during the days following ingestion of the bacteria, and that the presence of proliferation peaks appeared to correlate with more clinical symptoms and higher serum antibody response levels against TW11681 virulence factors. Further studies are needed to identify the underlying reasons for the lack of these proliferation peaks and if and how they relate to colonization and subsequent symptoms and immune response development. However, our results underline the need to monitor stool ETEC concentration during controlled human infection model studies to better explain variation in responses to the infection, and during human challenge vaccine trials to increase the accuracy of the protective efficacy estimates when testing new vaccine candidates against ETEC diarrhea.

## Methods

### Strain description

ETEC strain TW11681 (O19:H45; GenBank BioProject: PRJNA59749) was isolated in Guinea-Bissau in 1997 from the stool of a 6 month old girl who had diarrhea [10]. This ciprofloxacin-sensitive strain has genes encoding the STh and the two colonization factors CFA/I and Coli Surface antigen 21 (CS21). TW11681’s genome size is approximately 5.30 Mbp, around 316 kbp of which is plasmid DNA [17]. Phylogenetically, it belongs to an epidemiologically important ETEC lineage that is often found to be associated with childhood diarrhea (ETEC8 family [3] and Lineage L6 [4]).

### Controlled human infection model study

The volunteer study is based on experimental infection of nine healthy 23–28 years old students (1 man and 8 women) living in Norway who had not traveled to LMICs within 12 months prior to study start. In brief, the volunteers were included in the study in successive groups of three during the fall of 2016. The volunteers had fasted overnight before orally ingesting the dose at 11:00 a.m. at the Infectious Diseases Ward at Haukeland University Hospital, Bergen, Norway. They drank 120 ml 1.33% bicarbonate buffer, followed after 1 min by 30 ml of the bicarbonate buffer containing 1 × 10^6^, 1 × 10^7^, or 1 × 10^8^ CFUs of TW11681. The volunteers were allowed to drink and eat normally 1 h after that. The TW11681 doses were prepared by culturing the strain on animal product-free LB agar plates before harvesting, and the cells were re-suspended and subsequently washed three times in phosphate-buffered saline before being administered to the volunteers. Clinical signs and symptoms were self-reported by the volunteers during the daily clinical evaluations, stool samples were obtained daily, and blood samples were collected on the day of the infection and 7, 10, and 28 days after. Stool consistency was graded on a scale from 1 to 5, where the grades seen in this study were 1 and 2 (firm and soft formed stools, respectively) and 3 (viscous opaque liquid or semiliquid which assumes the shape of the container). The volunteers were considered to have diarrhea if they pass grade ≥ 3 stools with a combined weight of ≥ 200 g over a 48-h period or a single grade ≥ 3 stool of ≥ 300 g. To clear the infection, ciprofloxacin treatment (500 mg orally two times daily for 3 days) was started in the morning 5 days after they ingested the dose. None of the volunteers became ill enough to warrant earlier treatment.

### Microbiological detection of ETEC

Stool specimens were acquired daily starting on the first day after the dose had been ingested. The specimens were streaked onto *Enterobacteriaceae*-selective Lactose Agar (Haukeland University Hospital, Bergen, Norway), followed by over-night incubation at 35 °C. A sample was considered to be negative for ETEC if no *E. coli*-like colonies were seen on the plate. If colonies were present, a representative selection of colonies from the most confluent part of the plate was collected with a 1 µl inoculation loop, DNA was then extracted by boiling and centrifugation followed by testing the supernatant for the presence of the ETEC toxin genes by PCR as described earlier [13].

### Stool DNA purification

For this study, we designed a DNA purification method that enables extracting genomic DNA equally well from all stool microbes and minimizing the amount of co-purified PCR inhibitors while allowing for purification in a microplate format. Up to one stool specimen for each day from each volunteer was included in the study, including the day the volunteers ingested the dose. Stools were stored for up to 20 h at 4 °C before being mixed by stirring and a representative selection was stored aliquoted at − 70 °C. After thawing, around 50 mg of the specimen was suspended in 200 µl Lysis Buffer (100 mM Tris–Cl, pH 9.0, 40 mM EDTA, 1% SDS, 1 µg/µl Proteinase K) followed by incubation at 60 °C for 60 min with ~ 160 mg 1:1 mix of acid-washed 0.1 mm and 0.5 mm Ø glass beads in a 2 ml deep-well microplate covered with an aluminum foil seal for cold storage (Thermo Fisher Scientific, Waltham, MA). After a 2 × 1 min vigorous vortex of the sealed plate, 70 µl 6 M ammonium acetate was added to each well, followed by re-sealing and mixing with a gentle vortex, 20 min incubation at − 20 °C to precipitate SDS, a 60 s vigorous vortex, and a 6000×*g* centrifugation for 20 min at 4 °C in a Heraeus Multifuge X3 centrifuge fitted with a HIGHPlate 6000 Microplate Rotor (Thermo Fisher Scientific, Waltham, MA). Note that the integrity of the aluminum foil seal should be checked before vortexing, and that a fresh foil should be added after adding the ammonium acetate. Up to 200 µl of the resulting supernatant was incubated agitated in room temperature for 30 min in a Nunc 96-well Fritted Deep Well Plate (Thermo Fisher Scientific) that contained approximately 90 µl powdered polyvinylpolypyrrolidone (110 µm particle size; Merck, Darmstadt, Germany), 50 mg Amberlite-XAD4 (Merck), and 50 mg Dowex-1X8 (Merck) resins. Prior to adding the supernatants, the resin, which reduces the amounts of potential PCR inhibitors, had been individually distributed to wells where they subsequently soaked for 2–3 h in 200 µl Equilibration Buffer (75 mM Tris–Cl, pH 9.0, 30 mM EDTA, and 1.5 M ammonium acetate) before buffer removal by centrifugation at 800×*g* for 1 min. After incubation, the filtrates were collected by centrifugation into a 1.2 ml low-profile microplate, and nucleic acids were precipitated by adding 200 µl isopropanol followed by 20 min agitation at room temperature and centrifugation at 6000×*g* for 20 min at 4 °C. After pipette aspiration, 50 µl Digest Buffer (20 mM Tris–Cl, pH 8.0, 4 mM EDTA, 0.8 µg/µl RNAse A, 1.6 µg/µl Proteinase K, 20 mM TCEP, and 0.05% Tween-20) that had been prepared immediately before use was added while the DNA pellets were still wet, and the plate was incubated agitated for 30 min at 37 °C. A mix of 20 µl AMPure XP (Beckman Coulter Inc., Brea, CA) and 70 µl Binding Buffer (10 mM Tris–Cl, pH 8.0, 1 mM EDTA, 20% PEG 8000, 2.5 M NaCl, and 0.05% Tween-20) was added to each sample and mixed by pipetting, followed by transfer to a 350 µl round-bottom polypropylene microplate, incubation for ≥ 10 min at room temperature, and incubation on a microplate magnet for ≥ 5 min. After discarding the supernatant, the magnetic beads were washed by re-suspending them in 90 µl Binding Buffer before washing twice with 70% ethanol on the magnet. After 2 min air drying, 50 µl Dilution Buffer (10 mM Tris–Cl, pH 8.0, 0.05% Tween-20) was added to the beads, and, after 30 min incubation with agitation at 37 °C, the supernatant containing the DNA was stored at − 20 °C.

### qPCR oligonucleotides

A probe-based qPCR assay was developed for quantitating TW11681 bacteria. The qPCR primers were designed to target the *E. coli* O19-specific O-antigen polymerase gene (*wzy*, 1,179 bp; GenBank accession no.: LC223608.1), which is present as a single copy on the TW11681 chromosome. Gene sequences from 37 *E. coli* strains having > 90% sequence identity with *wzy* in TW11681, 35 of which had 100% identity, were downloaded from the whole-genome shotgun contigs database in GenBank and included in the alignment. The gene appears to have little sequence similarities with other known prokaryote and eukaryote sequences. The forward (GATGGTTAGTTTTATGACTGG; O19-wzy-TF) and reverse (GAAGAGACTAAGAACTTAGTTG; O19-wzy-TR) primers bind at gene nucleotide positions 941 and 1022, respectively, producing an 81 bp PCR fragment, and the probe (AGCACTCTTCTCGATTCCGACA; O19-wzy-TP), which binds at nucleotide 993, was labelled with 6-FAM (5′-end) and BHQ1 (3′-end). Primer3 [18] was used to test the suitability of the selected primer and probe sequences. The primers have 100% identity with the genes used in the above-mentioned alignment, except the forward primer has one mismatch in position 5 against *wzy* of O19 E*. coli* strain FCP1.

### qPCR assays

Immediately before running the qPCR assays, the purified stool DNA was quantified by using the QuantiFluor dsDNA System (Promega Corporation, Madison, WI) and diluted in Dilution Buffer to 1.0 and 0.1 ng/µl for use as template DNA in the qPCR assay. The assay was performed on four replicates, two for each template dilution, in white 384-well PCR plates on a LightCycler 480 machine (Roche Life Science, Penzberg, Germany). Each 9 µl reaction contained 1X ABsolute qPCR mix (Thermo Fisher Scientific), 0.4 µg/µl each of O19-wzy-TF and O19-wzy-TR primers, 0.2 µg/µl O19-wzy-TP probe, and 1.5 µl template DNA. The plates were incubated for 15 min at 95 °C, followed by 45 cycles of 20 s at 95 °C and 90 s at 60 °C.

The positive control DNA template was prepared from frozen aliquots of purified genomic DNA from three strains (TW11681, ETEC strain TW10722 [O115:H5], and *E. coli* laboratory strain BL21(DE3) [O7:H-]) that had been diluted in Dilution Buffer and pooled to a final concentration of 0.5 ng/µl of each strain. Tenfold dilutions ranging from 0.5 ng/µl to 50 fg/µl, as well as 25 and 12.5 fg/µl were used in triplicates as template DNA for positive controls in the assay, while Dilution Buffer was used for the corresponding no template controls. Since each TW11681 genome is 5.30 Mbp and, therefore, weights 5.7 fg, 1.5 µl of the positive control template dilutions added to the qPCR ranges from 132,000 to 3 TW11681 genome copies.

qPCR curves were analyzed by using the LightCycler 480 Software, version 1.5.1.62 (Roche Life Science), where the quantitation cycle (Cq) for each curve was determined by using the Second Derivative Maximum Method. The regression curve of the results from the positive control dilution series was generated by using the same software, and the linear dynamic range of the assay was subsequently set to span the dilutions that gave Cq results that closely conformed to this regression curve.

We tested the suitability of the qPCR annealing and elongation temperature by running the assay on a temperature gradient spanning 50–63 °C. When separated on a 4% agarose gel, a single distinct band corresponding to the expected 81 bp PCR product was seen for all reactions (data not shown), suggesting that 60 °C is suitable for the assay. We chose to use a long (90 s) annealing and elongation time to allow for more flexibility if multiplexing the assay with primers for additional qPCR targets.

To check for any negative effects of PCR inhibitors in the sample template DNA, we assessed whether the reactions containing 0.1 ng/µl template DNA tended to have higher PCR efficiencies than those containing 1.0 ng/µl template DNA. We calculated the mean Cq difference between the two replicates that were based on the 1.0 ng/µl template DNA and those that were based on the 0.1 ng/µl template DNA, excluding results that fell outside the estimated linear dynamic range of the assay. Histograms and Normal Q–Q Plots were then drawn to visually assess whether the distribution of these differences was normally distributed and symmetric, and Shapiro–Wilk Normality Test and Sample Skewness based on the traditional Fisher-Pearson Coefficient of Skewness test were performed to test these assumptions. We used the moments package [19] in R, version 3.4.2 [20] for these analyses.

### Immunological assays

Serum was prepared from blood collected 0, 10, and 28 days after the TW11681 cells had been ingested. We used multiplex flow-cytometry bead assays to measure IgA and IgG/IgM antibody responses to the two proteins CfaB (UniProtKB ID: P0CK93) and YghJ (UniProtKB ID: A0A080EY22). CfaB is the major structural protein of the plasmid-encoded fimbrial ETEC colonization factor CFA/I [5], while YghJ is a highly conserved enzyme involved in degrading the major mucins in the small intestine during ETEC colonization [21]. The genes encoding CfaB and YghJ, excluding the sequence encoding the signal peptides, were amplified by PCR and cloned into the pET-30 (Merck Millipore, Burlington, MA) expression vector. The 16 and 167 kDa, respectively, proteins were expressed in ClearColi BL21(DE3) cells (Lucigen, Middleton, WI) and purified by His-tag capture and dialysis against 20 mM phosphate buffer, pH 7.4 containing 0.3 M NaCl. The proteins were cross-linked to Cyto-Plex carboxylated polystyrene beads (Thermo Fisher Scientific) by using carboxyl-reactive chemistry, incubated with human volunteer serum and, subsequently, with fluorescently labeled anti-IgA or anti-IgG/IgM secondary antibodies (Jackson ImmunoResearch Inc, West Grove, PA). Each bead’s secondary antibody fluorescence intensity was measured on a flow cytometer, and the median intensity minus the median intensity of the negative control, which was beads labeled with His-tag labeled glutatione s-transferase, was used in the analyses.
